# Rethinking Sampled-Data Control for Unmanned Aircraft Systems

**DOI:** 10.3390/s22041525

**Published:** 2022-02-16

**Authors:** Xinkai Zhang, Justin Bradley

**Affiliations:** 1 Artificial Intelligence, Volvo Cars Technology USA, Sunnyvale, CA 94085, USA; 2 School of Computing, University of Nebraska-Lincoln, Lincoln, NE 68588, USA; justin.bradley@unl.edu

**Keywords:** resource-aware control, co-regulation, feedback scheduling, time-varying system

## Abstract

Unmanned aircraft systems are expected to provide both increasingly varied functionalities and outstanding application performances, utilizing the available resources. In this paper, we explore the recent advances and challenges at the intersection of real-time computing and control and show how rethinking sampling strategies can improve performance and resource utilization. We showcase a novel design framework, cyber-physical co-regulation, which can efficiently link together computational and physical characteristics of the system, increasing robust performance and avoiding pitfalls of event-triggered sampling strategies. A comparison experiment of different sampling and control strategies was conducted and analyzed. We demonstrate that co-regulation has resource savings similar to event-triggered sampling, but maintains the robustness of traditional fixed-periodic sampling forming a compelling alternative to traditional vehicle control design.

## 1. Introduction

The design of efficient, intelligent, and safe unmanned aircraft systems (UASs) is challenging, especially as onboard resources are stretched to maximize performance [1]. Emerging UASs are equipped with complex suites of computing (cyber) and mechatronics (physical) systems. They are expected to provide both highly varied functionalities, outstanding application performances, and remain safe, all within the available resources [2]. Computing and timing are key factors determining holistic system behavior [3,4] and, hence, must be first-class design parameters in such intelligent control systems. To optimize the use of computing resources—autonomy and control algorithms and associated resource requirements need to be considered simultaneously, or “co-designed” [5].

In this article, we focus on comparing the control performance of a multicopter UAS under different sampling strategies varying in the level of a “co-design” of computing resources (sampling rate) and holistic system performance. Specifically, we examine traditional fixed-periodic control [6], event-triggered control [7], self-triggered control [8], and a new hybrid sampling strategy we developed—“cyber-physical co-regulation” [9]. Cyber-physical co-regulation incorporates computational resource allocation alongside traditional control performance in a single model [9]. Cyber and physical controllers can then be co-designed to meet holistic performance requirements. The computational effector, sampling rate, is adjusted in response to off-nominal conditions in the controlled system, and the physical effector adjusts control outputs corresponding to the current (changing) sampling rate. To analyze the influences from computing and timing, the controllers are all designed based on a unified optimal control strategy—linear quadratic regulator (LQR). Our previous work focused on co-regulation design methodologies [10], co-regulated system stability analysis [11], co-regulated controller design [12], and proof-of-concept demonstrations of control and computing features of co-regulation design on a simple inverted pendulum system [13]. In this work, we focus on a detailed analysis of control and computing for UASs. We conducted a thorough comparison of control performances and computational efficiencies among different sampling strategies on an UAS to demonstrate how computing and timing can affect control. We highlight the unique benefits of our proposed co-regulation strategy on control performance, computational efficiency, and system robustness over the traditional fixed-periodic, event-triggered, and self-triggered controllers. We propose new evaluation metrics for analyzing UAS control and computing performances. We discuss the implementation of a co-regulation strategy to provide insight to control engineers on how to design co-regulated systems. Moreover, a more thorough discussion on the pitfalls of event-triggered and self-triggered sampling strategies on UAS is presented. Quantitative evaluations for all of these strategies were conducted based on evaluation metrics that could reflect both control performance and computing costs.

## 2. Motivating Example

In a traditional fixed-periodic, computer controlled system, the control laws and their associated sampling or execution rates are intrinsically linked [6]. The sampling rate is typically overdesigned—selected to account for the worst-case anticipated noise and response characteristics, giving the system a safety margin [14]. The behavior of the control loops, and the relation between the control performance and controller execution rate, such as the results in [15], suggest that static resource allocation may not be optimal for system performance when computing resources are limited. Intuitively, a controller of a plant operating close to its equilibrium may only require a much lower sampling rate than a plant operating far from its equilibrium point [16]. In size, weight, and power constrained vehicle systems running control tasks and a myriad of other tasks, such as perception, learning, planning, and data processing—redistributing these computing resources at runtime is key to maximize the overall system performance [15,16].

**Figure 1:**
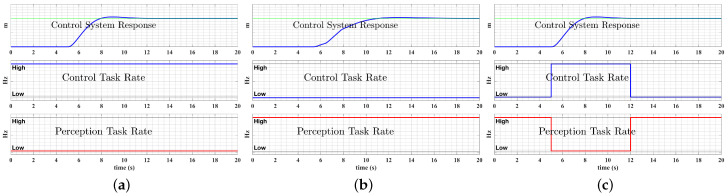
Different resource allocation strategies for UAS (example response for illustration purposes). (**a**) static high-rate control with low-rate perception; (**b**) static low-rate control with high-rate perception; (**c**) dynamic resource allocation for control and perception tasks.

Figure 1 illustrates three possible resource allocation strategies for a simplified surveillance multicopter in which a flight control task and a perception task are executed concurrently and share a fixed amount of computing resources. The plots depict an example response from the simulation for illustration purposes. The flight control task in this example is for the UAS to track a series of waypoints and the control system response in each subfigure depicts the cross tracking error with respect to the reference waypoint in meters. Assume that the processor has a limited amount of resources that can allow only one task to run at a higher rate and the other at a lower rate during runtime. The lower rate and higher rate tasks respectively correspond to lower and higher resource consumption. In typical priority-driven scheduling, the highest priority task is given system resources—CPU cycles [17]. A task’s priority, in most safety-critical systems, is dependent on the task’s period, particularly for hard real-time tasks, such as control and perception [17].

Figure 1a,b show two static (fixed-rate) resource allocation strategies; Figure 1c shows a simple dynamic (variable-rate) resource allocation policy. In Figure 1a, the control task is consistently executed at a high rate, while the perception task is executed at a low rate because of the limited computing resources. This leads to a good control task performance, but potentially poor perception performance. In contrast, in Figure 1b, the control system performance deteriorates because a major portion of the computing resources are allocated to the perception task. The control task has to be executed at a low rate; thus, the system response becomes slow. In Figure 1c, the resources are dynamically allocated, in a closed-loop fashion, according to plant dynamics and performance to the control and perception tasks. The benefits of this dynamic resource allocation are that it has good control performance *and* efficient and effective resource usage. Thus, the system computational resources are efficiently allocated to different tasks in a closed-loop fashion, which can increase the holistic system performance.

**Figure 2:**
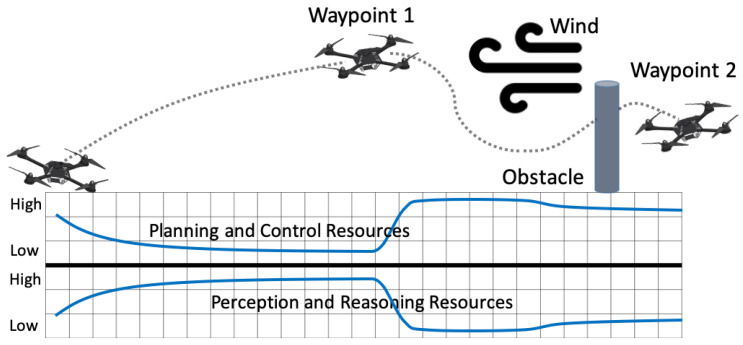
Dynamic resource reallocation of a UAS.

Figure 2 provides an example of this dynamic resource allocation design on a UAS. When flying from an initial position to waypoint 1, in a dynamically quiescent environment, the planning and control task should consume fewer resources as the UAS moves closer to its target (less trajectory tracking error). The resources are more efficiently applied to improve perception, reasoning, or data collection activities. In contrast, when flying from waypoint 1 to waypoint 2, the UAS should “pay more attention” (i.e., reallocate resources) to the planning and control task to navigate itself to the target while avoiding obstacles in the congested environment. During this time, higher level reasoning and perception tasks can wait, while resources are reallocated to control, planning to improve tracking performance.

To enable a resource-aware controller capable of adjusting performance and resources as needed, we developed a feedback control-based model for controller tasks, in which computing resources and control performances are jointly considered. The co-regulated systems are capable of dynamically reallocating resources to control tasks based on system states in a closed-loop fashion, while the control task could provide an adjustable performance depending on the dynamically reallocated resources at runtime. In this paper, we conduct a comparative experiment based on a multicopter UAS to analyze the computational and physical control performances of co-regulation and the conventional fixed-periodic, event-triggered, and self-triggered sampling and control strategies. Results show that the co-regulated UAS preserves the resource savings similar to event- and self-triggered control strategies, but maintains the high-quality control performance and system robustness as a fixed-periodic control strategy.

## 3. Background

Here, we provide a brief overview of dynamic resource allocation and its relationship to intelligent control. We then narrow down to resource allocation in control systems, focusing on how computational resources, in the form of a sampling rate, have traditionally been allocated.

### 3.1. Dynamic Resource Allocation

Although dynamic physical resource allocation in the form of mutable or reconfigurable structures has been studied, we focus on dynamic cyber resource allocation (i.e., CPU time, memory). This approach offers system adaptability to available resources when performance requirements of a running application are changed, or the current allocation is not sufficiently close to optimal [18]. Strategies, such as voltage and frequency scaling CPUs [19], dynamically adjust CPU clock rates, thereby adjusting the actual physical CPU time any task gets. A side-effect of these strategies is a corresponding change in power consumption. Rate-adaptive and rhythmic tasks [20] adjust the CPU schedule to obtain a similar result leaving remaining CPU cycles to be reallocated elsewhere, thereby forfeiting power savings. A framework called “time bands”, in contrast, reallocates tasks to different levels of time granularity [21]. To know when, and how much to adjust resources, the above must be connected to a measure of “quality of service” and other performance metrics [22]. This was addressed most recently by applying reinforcement learning to design an optimal resource allocation system [23].

These methods have largely remained divorced from the specifics of control and UAS, or otherwise do not incorporate such systems into their models. In contrast, most recently, attention has been given to building formal resource architectures and controllers for computer systems utilizing knowledge gained in the control community, specifically for robust control [24]. Our work complements and enhances this work, but takes the approach of directly integrating computational resources into control-specific models, allowing for the co-design of both computational and physical controllers.

### 3.2. Overview of Sampling Strategies

Controllers are designed to meet performance specifications for the system and are executed on a digital computer. In this paradigm, allocating CPU cycle/time takes the form of different sampling strategies resulting in the sampled-data control class of systems [14]. If we consider the sampling period as a control variable, the predominant design amounts to open-loop control of periodic execution, or fixed-periodic sampling. The period is typically chosen according to worst-case conditions, leading to inefficient implementations in terms of processor usage, communication bandwidth, energy, etc. As in the UAS example, executing the control tasks are under a fixed period, when states and the environment are quiescent waste computational resources that could be used for reasoning, decision-making, or adaptation.

The inefficient allocation of resources in fixed-periodic sampling motivates research in aperiodic sampling strategies [13]. Aperiodic sampling in the control is exemplified by event-triggered control where control actuation instances are performed when needed [13]. In event-triggered control, a control cycle is only executed when the triggering condition is violated [8]. These aperiodic sampling strategies can greatly conserve computational resources, but suffer from the disadvantages of event-triggered systems. The triggering condition needs to be continuously monitored and, thus, more sampling instances may be required. Moreover, the cases where trigger conditions are not met cannot be distinguished from failure in detecting/communicating the event [13,17]. This reduces the robustness of event-triggered control strategies in dynamical environment and network conditions, and makes developing a mathematical foundation for this class of controllers challenging [13,25].

**Figure 3:**
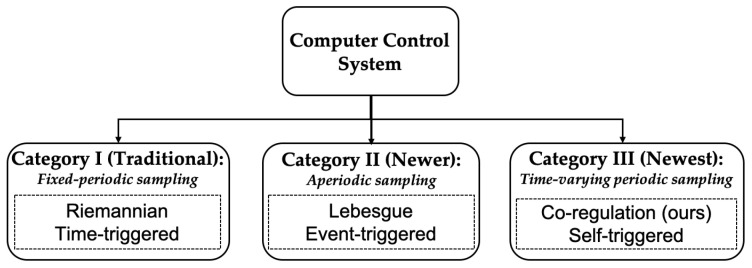
Three categories of control systems.

The research in self-triggered control provide a new type of sampling strategy, time-varying periodic sampling [13]. Time-varying periodic sampling achieves the benefits of periodic methods in terms of design, but because the sampling period changes, it conserves computational resources similar to aperiodic control strategies [13]. In self-triggered control, the time for next sampling and control instance is precomputed during the current control cycle using previously received data and knowledge of system dynamics [8]. In [9], we introduced a new hybrid time-varying periodic sampling strategy that can dynamically vary the sampling period at runtime, depending on computing demands and system state feedback. This allows computational and physical resources to be dynamically reallocated as needed. Therefore, going beyond the traditional classification of Riemann and Lebesgue sampling approaches in [25], we reclassify computer control systems into three categories: fixed-periodic sampling, aperiodic sampling, and time-varying periodic sampling, as shown in Figure 3.

## 4. Control under Different Sampling Strategies

Consider a linear control system model in a general form,

(1) $$\begin{array}{cc} \dot{x} & =Ax+Bu. \end{array}$$

where $x\in R^{n}$ is the state and $u\in R^{m}$ is the control input. We introduced the control design for each of the sampling categories in Figure 3 based on this general model. To analyze the performance of different sampling strategies, we employed a unified discrete linear quadratic regulator (DLQR) algorithm for fixed-periodic, event-triggered, self-triggered, and a co-regulated controller design.

### 4.1. Category I—Fixed-Periodic, or Time-Triggered Control

For a fixed sampling interval $T_{d}$, we can discretize the system model (1) as

(2) $$\begin{array}{c} x[k+1]=\Phi x[k]+\Gamma u[k]. \end{array}$$

For the common state-feedback control [14], the control input can be denoted as u[k]=−Kx[k], where the control gain *K* can be designed to meet the performance criteria. For the DLQR algorithm, the control gain, *K*, can then be decided by choosing appropriate Q and R matrices.

### 4.2. Category II—Event-Triggered Control

Event-triggered control consists of two elements—a feedback controller that computes the control input and a triggering mechanism that determines when the control input has to be updated again [8]. To implement event-triggered control in a computer, the controller needs to sample the system at a fixed base-period $T_{d}$ (internal sampling interval) to decide if a new control update is needed [26]. Generally, a triggering parameter needs to be designed to execute a control instance to guarantee system stability. This triggering parameter should be designed depending on the system model. In this paper, we have adapted the event-triggered controller in [27] to our multicopter UAS and used it as the basis for results comparison. This algorithm updates the control signal once the UAS states deviate more than a certain threshold from a desired value.

### 4.3. Category III—Self-Triggered Control

Event-triggered is reactive; it requires constant monitoring of a triggering condition. Self-triggered control, on the other hand, is proactive and computes the next sampling and control instance ahead of time [8]. At each sampling instant, the control signal values, as well as the next sampling instance time, are both calculated based on the current state. To implement self-triggered control in a computer, an inter-execution time $T_{d}$ is required to work as the time span unit that, when combined with the calculated next control time step $k^{*}$, the exact time for the next sampling and control instance can be achieved as $k^{*}T_{d}$. Similar to event-triggered control, a triggering parameter is needed to decide the appropriate next control instant to guarantee system stability. In our comparison test, we leverage the self-triggered control algorithm in [27]. This algorithm determines the subsequent control updates based on the prior ones, obviating the necessity for continual measurement error monitoring [13].

### 4.4. Cyber-Physical Co-Regulation

Cyber-physical co-regulation is a time-varying periodic sampling and control strategy, it can adjust system performance by simultaneously co-regulating the control input and its required computing resources. We augmented a traditional state space control model as in Equation (1) with a model of the computational control task. This resulted in an augmented, stacked state-space system model

(3) $$\begin{array}{cc} \; & \dot{x}=Ax+Bu\\ \; & {\dot{x}}_{c}=u_{c} \end{array}$$

where *c* denotes “computational”. The term “computational” refers to the state of the resource, and in this case, $x_{c}$ refers to the physical system’s control task execution rate, which is regulated by the computational control input $u_{c}$.

**Figure 4:**
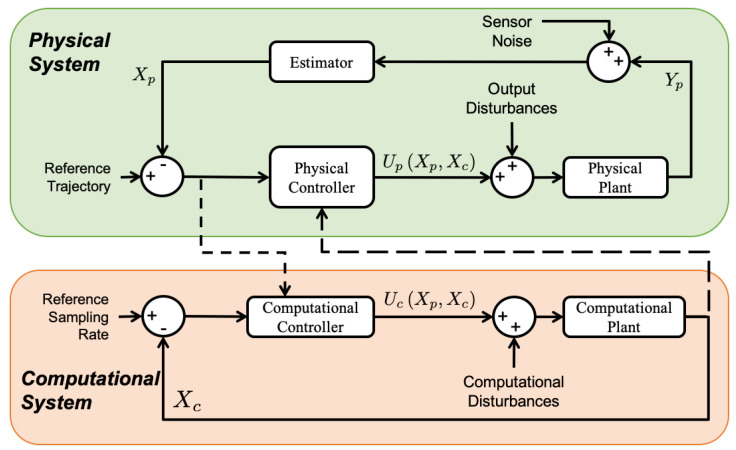
Co-regulation block diagram [10].

Figure 4 shows the co-regulation method developed for the multicopter UAS control in our previous work [10]. The computational system monitors the physical state error at runtime and dynamically reallocates computational resources (i.e., sampling rate) in reaction to physical performance. When the physical state error increases, the computational controller increases the sampling rate; when error decreases, the sampling rate decreases [13]. The physical system then executes the control task and adjusts the physical system performance according to the time-varying sampling rate. The physical controller is designed to provide performance guarantees for the UAS when working under the time-varying sampling rate.

Because of the dynamically changing sampling rate at discrete intervals, the discrete system matrices Φ and Γ are not static and need to be recalculated at each time step *k*. The resulting discrete-time-varying system model is then [13],

(4) x[k+1]=Φ[k]x[k]+Γ[k]u[k]

and the control input is

(5) u[k]=−K[k]x[k]

The controller is designed using a sequence of DLQR control gains for a sequence of sampling rates [14]. The control gains are mapped with different sampling rate values, and are then deployed corresponding to the current sampling rate [13]. This control design is referred to as a “gain-scheduled DLQR (GSDLQR)” and is described in Table 1 [10,12], with stability results demonstrated in [11]. In this work, we primarily focused on a comparative analysis of the control and computing features among different sampling strategies. Although advanced methods for UAS nonlinear control [28] exist, we leveraged a unified linear DLQR control algorithm for different sampling strategies to simplify the control design and highlight the performance differences brought by different sampling strategies.

Co-regulation, as with event- and self-triggered strategies, needs to know when to take the next sample [13]. We structured this as a feedback computational controller that calculates the coupled control input $u_{c}$, which dynamically modifies the sampling rate in response to the system’s dynamics. In prior work [10], we defined a control law for computational systems as

(6) $$\begin{array}{c} u_{c}\left[k\right]=K_{cp}\left(x\left[k\right]-x_{ref}\left[k\right]\right)-K_{c}\left(x_{c}\left[k\right]-x_{c,ref}\left[k\right]\right). \end{array}$$

In response to physical state error, the coupling gain $K_{cp}$ was employed to increase the sampling rate of the system. The gain $K_{c}$, conversely, directs $x_{c}$ in the direction of the desired reference sampling rate $x_{c,ref}$. We applied an optimization approach introduced in [10] to determine values for the gain values $K_{cp}$ and $K_{c}$. As a result, the discrete-time computational system model for the current sampling instance *k* can be denoted as [13]

(7) $$x_{c}\left[k+1\right]=x_{c}\left[k\right]+\frac{1}{x_{c}\left[k\right]}u_{c}\left[k\right].$$

Thus, based on the current state of the plant, the next sample instance time can be determined using Equations (6) and (7).

To implement the co-regulation strategy in the software, we needed to add constraints to the computational system model (7) to limit the sampling rate values, $x_{c}$, to a set Σ that contained a finite number of possible values. $\Sigma =\left\{f_{1},f_{2},\ldots ,f_{N}\right\}$ is a pre-defined finite set that contains stable sampling rate values as prescribed operating points. This limits the sampling rate of the co-regulated system to a finite number to simplify the analysis. The bounds and the resolution of the values in Σ can be customized depending on the application. The general rule to generate Σ is to:

1. Set the upper bound based on the system computational bandwidth given all other computing tasks;
2. Set the lower bound to the rate where system performance degrades beyond acceptable limits, or otherwise is unstable;
3. Set the resolution based on the system dynamics and application scenarios, which can guarantee system stability and accommodate performance requirements, such as disturbance rejection, dynamic response, etc.

In the software implementation, the GSDLQR gain matrices are saved as a look-up table. In each control cycle, the appropriate DLQR gain matrix that maps with the current sampling rate is leveraged to compute the control signal. Then the next sampling rate value is calculated by the computational control model based on the current state feedback. The control task execution rate is decided by the computational controller based the the real-time state feedback. The software implementation of the co-regulation algorithm is summarized in Algorithm 1.
**Algorithm 1:** Cyber-physical co-regulation. **Result**: Physical control input u[k]      Control task rate $x_{c}\left[k+1\right]$ **Input:**
$x\left[k\right],x_{ref}\left[k\right],x_{c}\left[k\right],x_{c,ref}\left[k\right],K_{cp},K_{c},\Sigma$
 **Output:**
$u\left[k\right],x_{c}\left[k+1\right]$ **while**
*Algorithm is running*
**do** 
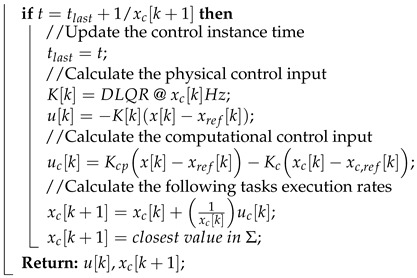



The novelty of the co-regulation approach is in its coupling of computational and physical systems via equations of motion rather than incorporating the delays of motion into the models used for task scheduling. That is, at the feedback control level, computational and physical resources are balanced dynamically rather than at a higher planning level [9]. Co-regulation does not replace traditional planning, but supplements it by reactive reallocation of resources within the reference trajectories commanded by the planner [9].

Co-regulation, conceptually, can be applied to a wide variety of control problems, including nonlinear controllers, and there are currently two main approaches to designing them. The most widely applicable are methods such as the presented gain scheduling framework that are generalizable to virtually any control strategy as long as multiple sampling-rate-targeted controllers can be designed. In this paradigm, co-regulation becomes a switched system with associated performance guarantees [12]. More generally, however, smoother, more robust controllers can be developed as long as a single control law that works for a wide range of sampling rates can be found. As an example, for attitude control of a CubeSat, we introduced a control strategy based on propagating the Riccati equations forward in time (rather than the traditional "backward in time") [9]. In that paradigm, the controller evolves alongside the sampling rate and discrete-time-varying dynamics. Such strategies are more difficult to design since they rely on discrete-time-varying dynamics (due to the time-varying sampling rate) and developing such control laws is an active area of research. Minimum sampling rates to ensure good performance are highly dependent upon the speed of the system dynamics, and experimentally, we observed that single co-regulated control laws tend to evolve more slowly without an associated sampling rate prediction mechanism or trajectory. As a result, our existing controllers of this type are applied to systems with slower dynamics (e.g., CubeSat). In contrast, the presented gain scheduling paradigm, due to its switching nature, is better applied in systems with faster dynamics. Eventually, we envision an all-encompassing framework for designing co-regulated controllers dependent upon the speed of the system dynamics, expected computational capabilities, expected computing task sets, and resource requirements.

## 5. Evaluation Metrics

We intend to quantify both physical and computational characteristics of the system in order to more holistically evaluate system performance. Physical evaluation metrics primarily assess performance of the plant’s response to references and the actuation effort required to achieve it. Computational evaluation metrics primarily assess the computational resources allocation for control task, which include the number of sampling and control instances during the test cycle.

### 5.1. Physical Evaluation Metrics

Our first metric is the time averaged square of physical state error (PSE),

(8) $$\mathrm{PSE}=\frac{1}{t_{tot}}\sum_{i=0}^{n}{\left(x_{i}-x_{ref}\right)}^{2}\;t_{i}.$$

This metric provides an all-encompassing look into how well all states are being regulated. Because average error does not address control inputs that may saturate actuators, we introduce an additional metric, maximum state error (MSE), which evaluates the maximum deviation of the plant states from the reference,

(9) $$\mathrm{MSE}=\max{\left(x_{i}-x_{ref}\right)}^{2}.$$

For control effort, we introduced a metric capturing the time weighted average of the square of the control input (i.e., control effort (CE)),

(10) $$\begin{array}{c} \mathrm{CE}=\frac{1}{t_{tot}}\sum_{i=0}^{n}u_{i}^{2}\;t_{i} \end{array}$$

where $t_{tot}$ is the total simulation time (in seconds), $t_{i}$ is the length of time for the *i*th simulation step, *n* is the total number of simulation steps, $u_{i}=u\left[k\right]=const.\;\mathrm{on}\;kT_{d}\leq t<\left(k+1\right)T_{d}$. This metric provides an indicator of energy and power usage. We also introduced a metric to quantify the energy cost W for the UAS in one entire flight test process. The power required to produce a given thrust is $P=\sqrt{\frac{T^{3}}{2\rho A}}$, where *T* is the rotor thrust, *A* is the area of the spinning propeller, and ρ is the air fluid density [10]. Then the energy cost W in the whole process can be denoted as

(11) $$W=\frac{1}{t_{tot}}\sum_{i=0}^{n}P\;t_{i},$$

as an additional way to evaluate the controller efficiency.

### 5.2. Computational Evaluation Metrics

On the computational side, we evaluated the sampling and control task resource utilization by counting the number of sampling instances and control instances, respectively, during the test time. Let $k_{sample}$ represent the execution cycle (time index) of the sampling task, incrementing each time the sampling task runs. We introduce the sampling computational time (SCT) metric as

(12) $$\mathrm{SCT}=k_{sample}.$$

Similarly, we define the control computational time (CCT) metric as

(13) $$\mathrm{CCT}=k_{control},$$

where $k_{control}$ represents the execution cycle (time index) of the control task.

## 6. Results

In this section, we deploy different sampling strategies on a multicopter UAS nonlinear simulation and compare the resulting flight control performances and resource consumption. The UAS state consists of the vehicle’s position ${\left(x,y,z\right)}^{T}$, velocity in $R^{3}$, orientation in roll (ϕ), pitch (θ) and yaw (ψ) angles, and angular rate of change in yaw [12]

$$x_{p}={\left(x,y,z,\phi ,\theta ,\psi ,\dot{x},\dot{y},\dot{z},\dot{\psi }\right)}^{T}.$$

A lower-level attitude controller was integrated with the UAS, which accepts the desired thrust (*T*), roll angle (ϕ), pitch angle (θ), and body yaw rate (*r*) as inputs [12]. Thus the control input for the UAS is $u_{p}={\left(\phi ,\theta ,r,T\right)}^{T}.$ The equations of motion of the UAS model are

$$\left[\begin{array}{c} {\ddot{p}}_{n}\\ {\ddot{p}}_{e}\\ {\ddot{p}}_{d} \end{array}\right]=-\frac{T}{m}\left[\begin{array}{c} \cos\phi \sin\theta \cos\psi +\sin\phi \sin\psi \\ \cos\phi \sin\theta \sin\psi -\sin\phi \cos\psi \\ \cos\phi \cos\theta \end{array}\right]+\left[\begin{array}{c} 0\\ 0\\ g \end{array}\right]$$

where *m* is the total mass of the UAS, and *g* is gravity [12]. These nonlinear equations of motion were employed to build the high-fidelity UAS flight simulation. The controllers were designed using a linearized state-space system model, such as (1), by linearizing the model at a stable hover equilibrium point. To compare and analyze the control performances and resource consumption among different sampling strategies, we developed fixed-periodic, event-triggered, self-triggered, and co-regulated controllers for this UAS and conducted the tests in unified environments. We leveraged the nonlinear equations of motion of the UAS to build this high-fidelity test environment to simulate the UAS performances when operated under different strategies.

### 6.1. Simulation Setup

The comparison test was conducted in a simulation environment that was built in Matlab R2017a on a 2.3 GHz Intel i5 processor computer. The control and computational performances of the UAS were recorded and quantified using the evaluation metrics in Section 5. The UAS parameters are specific to the "Ascending Technologies Hummingbird" and are listed in Table 2. More detailed specs of the UAS can be found in [29]. To study the effects of computing and timing, the fixed-periodic, event-triggered, self-triggered, and co-regulated controllers were all designed based on a unified optimal DLQR control algorithm with unified parameters. The controller parameters *Q* and *R* were manually tuned, and are shown in Table 2. The selection of computational gain $K_{cp}$ and $k_{c}$ parameters for co-regulation are based on the optimization scheme in [10] that targets minimizing a cost function composed of terms measuring resource usage, control performance, and energy consumption.

The sampling rate of the fixed-periodic control was set to 10Hz as it could provide a sufficient control performance for this multicopter UAS. The internal sampling interval $T_{d}$ for event- and self-triggered control was set to 10Hz, the resulting discrete system model and control gain matrix were the same as the fixed-periodic control. The triggering parameters for this multicopter UAS model were manually tuned as σ=0.9 (event-triggered control) and μ=0.6 (self-triggered control) based on the system response to perform the “best” trade-off among resource utilization and control performance. The selected triggering parameters were located in the range where the system stability for the multicopter UAS model could be guaranteed [27]. For co-regulation, the reference sampling rate $x_{c1,ref}$ was set to 1Hz as the minimum resource we allocated to the control task. The time-varying control gain K[k] was calculated at runtime to provide performance guaranteed control.

### 6.2. Test Results

We compared the system performances of fixed-periodic control, event-triggered control, self-triggered control, and co-regulation in a UAS waypoint in the following scenario. In the first test, we set a single reference waypoint for the UAS to test the step response. The initial state of the UAS was set to a stable hover at one meter above the origin of the inertial frame, the single reference waypoint was set to two meters away in the *x* direction. Each test was timed for a total of 15s, and we manually added a wind disturbance of 0.2N in the positive *x* direction from 7 to 9 s.

**Figure 5:**
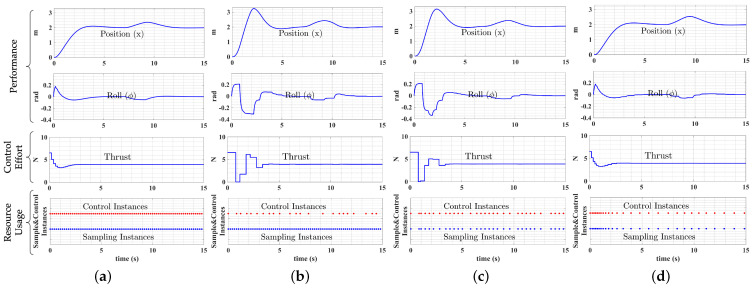
Performance comparison of fixed-periodic, event-triggered, self-triggered control, and co-regulation. (**a**) fixed-periodic DLQR; (**b**) event-triggered control; (**c**) self-triggered control; (**d**) co-regulation.

In this work, we assumed the magnitude and direction of the wind disturbance was constant to simplify the analysis. In an extended trajectory with varying disturbances, the system will adapt as “error” from the controller increases. The results are depicted in Figure 5, and quantified by evaluation metrics in Table 3. The “Position (x)” in Figure 5 depicts the UAS moving trajectory in the *x* direction in meters, which reflects the UAS flight control performance. Those transient responses in “Position (x)” that have smaller deviations from the reference (2 m) exhibited better performances. The controllers were designed as waypoint followers, which took position information as references. Waypoint following is the most ubiquitous type of multicopter control strategy in deployment.

The system performances from different sampling and control strategies are compared and depicted in Figure 5. In general, fixed-periodic control provides the “gold standard” of control performance but also utilizes the most computing resources [13]. Thus, a performance that is closer to the fixed-periodic controller generally indicates better control. The number of sampling and control instances reflects the resource consumption. Higher computational efficiency is exemplified by sparser instances. When compared with fixed-periodic control (Figure 5a), event-triggered control (Figure 5b) can save computational resources at the cost of significantly degraded control performance. The event-triggered controlled UAS has a 20% longer settling time and a 60% higher overshoot than when being controlled by the fixed-periodic controller. For the self-triggered control performance (Figure 5c), more computational resources can be saved since both sampling instances and control instances are reduced. In terms of physical control performance, self-triggered control has a similar settling time to event-triggered control, but has a 10% lower overshoot.

The co-regulated controller (Figure 5d) provided the best balance of control and computational performances among all sampling strategies. On the control side, co-regulation could achieve a nearly identical physical control performance compared with the fixed-periodic control, which retained significant advantages over event- and self-triggered controllers. On the computational side, co-regulated control required the fewest sampling instances, providing the greatest computational efficiency. In this test, the fixed-periodic, event-triggered, self-triggered, and co-regulated controllers all responded similarly to disturbances. Uniquely, however, when a disturbance occurred, the sample rate of the co-regulated controller quickly increased, allowing the system to promptly respond to the state deviation induced by the disturbance and maintain robust performance.

In Table 3, we show quantified results for the comparison test based on evaluation metrics. The results are normalized to provide a more straightforward comparison. The physical control performances best illustrate the PSE and MSE metrics; fixed-periodic control and co-regulation can provide approximately the same level of control performances, significantly better than event-triggered and self-triggered strategies. The control performances in the fixed-periodic control and co-regulation also lead to better energy efficiency presented by lower CE and lower W. The computational system performances are best illustrated by the SCT and CCT metrics. In all cases, the decrease of SCT and CCT metrics denoting less computational resources were allocated to the sampling and control task. The fixed-periodic DLQR controller consumed the most computational resources among all strategies. The event-triggered and self-triggered controllers could perform considerable savings in computing resources as a trade-off for degraded physical control performances. Event-triggered control consumed the least control instances during this test process. However, since it required consistently monitoring of the system states, the computational resource consumption for state sampling was the highest. Co-regulation consumed the least resource in sampling, and the physical control performance was far better than event-triggered and self-triggered strategies. From the results, co-regulation provided the most significant computing resources savings with minimal loss of physical performance. That is, the co-regulated system could achieve an (approximately) identical physical system performance as the fixed-rate controllers while saving significant computational resources similar to event-triggered and self-triggered controllers.

**Figure 6:**
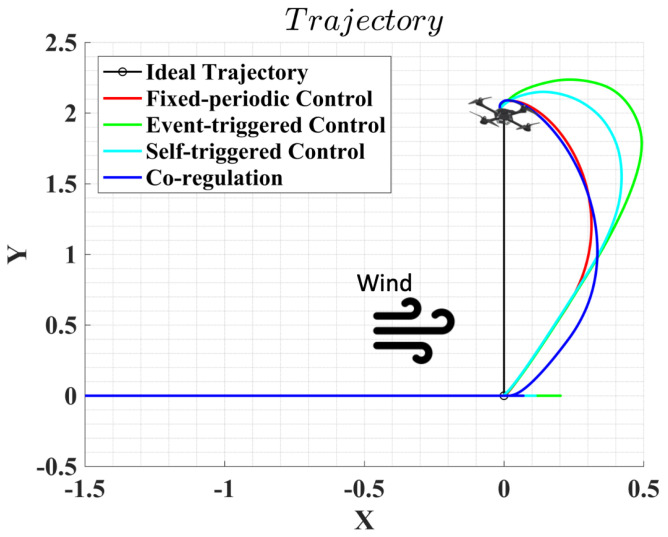
Waypoint following performance under disturbances.

Figure 6 demonstrates a top view of the UAS waypoint following performance under disturbances. This can provide a more straightforward comparison of how the UAS responds to disturbance under different sampling strategies. Two consecutive reference waypoints are set to 1 m away in the *x* direction, then 2 m away in the *y* direction. A disturbance of 0.3N is set in the positive *x* direction when the UAS moves towards the second waypoint. The results show that all four sampling strategies can provide robust performances against the disturbance. However, fixed-periodic control and co-regulation show better robustness to disturbances as the trajectory deviation caused by disturbances are smaller than event-triggered and self-triggered controllers.

## 7. Discussion

The primary benefits of the co-regulation strategy in computing resource saving and physical control performance were discussed, based on the comparison test results. Moreover, the proposed co-regulation design has some unique advantages, from the design prospective, when compared with event-triggered and self-triggered control strategies. It can overcome the disadvantages of not being able to distinguish lack of new information from detection/communication failures in an event-triggered control. For self-triggered systems, the only information provided at each time step is the next sampling instance. Co-regulation has the advantage of knowing precisely when the next sampling instant will be unless it is changed. That is, in the absence of a computational control law, it reverts to a fixed-rate control—a strong robustness characteristic.

**Figure 7:**
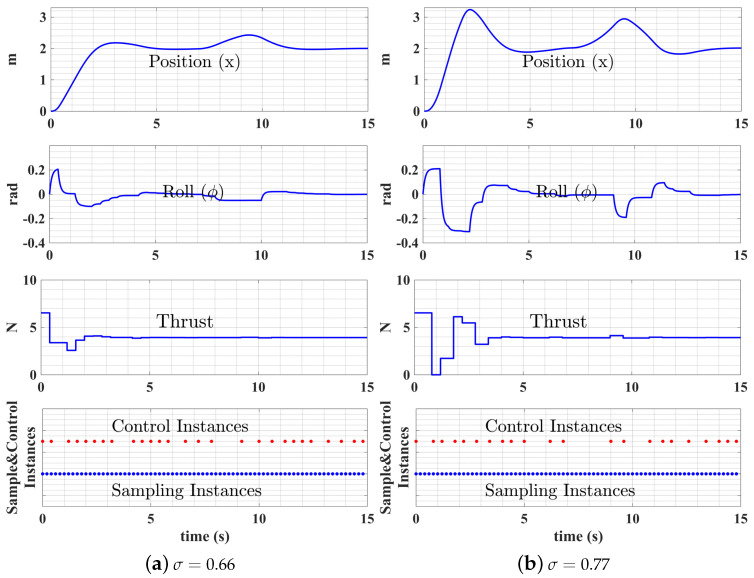
Event-triggered control performances with different σ values.

Event-triggered and self-triggered control algorithms add additional tuning and triggering parameters, which can bring extra uncertainties in the control performance. Thus, the system robustness can be reduced when compared with fixed-periodic and co-regulated controllers. During the test process, we found event-triggered and self-triggered control algorithms are very sensitive to parameter changes; that is, a tiny change in the triggering parameter can lead to huge changes in system performance. The UAS step responses under different triggering parameter values are recorded. Figure 7a,b, shows the UAS performance difference when the triggering parameter (σ) value is changed from 0.7 to 0.9, the resulting number of control instances decreases from 27 to 22, but the control performance is dramatically changed.

**Figure 8:**
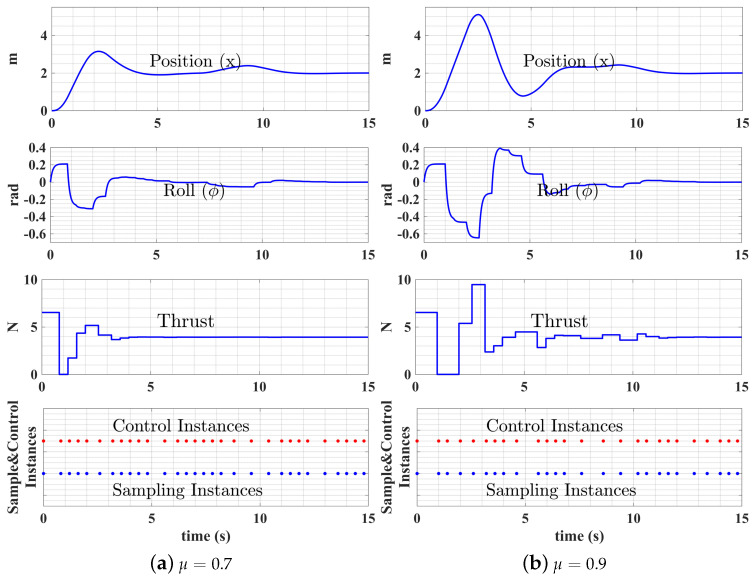
Self-triggered control performances with different μ values.

In self-triggered control, the system performance is even more sensitive to triggering parameter changes. Figure 8a,b shows the UAS performance difference when the triggering parameter μ value changes from 0.7 to 0.9, the resulting number of control instances decreases from 30 to 26, and the control performance is greatly affected.

The performances of event/self-triggered controllers can become more robust when we select more conservative triggering parameters. However, the sampling and control instances will increase to approximately the same level as a high-rate fixed-periodic controller, which will lose the advantages in conserving computing resources. On the other hand, less conservative triggering parameters of event-triggered and self-triggered controls can lead to more savings of resources at the cost of decreased system robustness. Co-regulation, as opposed to event/self-triggered control strategies, can provide much more robust and consistent system performances when we change either *Q* and *R* parameters for the physical system controller or $K_{cp}$ and $K_{c}$ parameters for the computational system controller.

Recent research studies have provided more advanced designs for event-triggered and self-triggered controls, where better trade-offs among resources and performances could be achieved. An important issue surrounding event-triggered and self-triggered controls is the complexity of the algorithms used for the online implementation that decides the sampling interval [30]. Such complex calculations can be challenging to real-time implementations, especially for applications where fast system dynamics are required. However, the co-regulation scheme calculates the sampling rate trajectory by a separate computational controller that can be executed extremely fast (complexity O(1)) with negligible resource consumption, such as a PID controller [9]. The physical controller can also be customized depending on different application requirements. The proposed GSDLQR algorithm for co-regulated systems can be implemented as a lookup table, which can be executed fast enough to meet real-time requirements in highly dynamic applications [10]. By detaching the computational and physical system, co-regulation can provide a higher degree of freedom for system design, as well as a highly robust framework that can provide performance guarantees for different application scenarios. Though discussed in the context of UAS in this work, co-regulation design is broadly applicable to cyber-physical vehicle and robotics systems, particularly those where careful allocation of resources is desirable (e.g., size, weight, and power constrained vehicles). More detailed information of a co-regulated control algorithm [10,12] and co-regulated system stability analysis [11] can be found in our previous work. Our future work will focus on extending the co-regulation design to different vehicle and robotic systems.

## 8. Conclusions

In this paper, we analyzed the trade-offs between computing resource utilizations, represented by sampling rate and control performance, to further explore an opportunity for more intelligent design strategies for UAS, to accomplish more with fewer resources. We conducted a comparison test of different sampling strategies to demonstrate how computing and timing can affect control performance. We highlighted the benefits of our proposed cyber-physical co-regulation strategy in overcoming the drawbacks of event-triggered and self-triggered controllers, while saving far more resources compared with traditional fixed-periodic strategies.

To make UAS more intelligent, they need the ability to adjust behavior (performance) *and* resources to meet increasing demands in uncertain environments. This type of rethinking of the foundations of autonomy is required to build the next generation of autonomous robotic systems. Co-regulation does exactly this, and can form the core of a new intelligent control system to meet this challenge. We anticipate this will make autonomous robotic systems more efficient, robust, and capable of adapting to changes.

## Figures and Tables

**Table 1:** Scheduled Gains at different sampling rates [10].

Rate $x_{c}$	Gain K[k]
$x_{c}=x_{c,\min}\;\mathrm{Hz}$	$K\left[k\right]=\mathrm{DLQR}\;@x_{c,\min}\;\mathrm{Hz}$
$x_{c}=10\;\mathrm{Hz}$	$K\left[k\right]=\mathrm{DLQR}\;@10\;\mathrm{Hz}$
$x_{c}=11\;\mathrm{Hz}$	$K\left[k\right]=\mathrm{DLQR}\;@11\;\mathrm{Hz}$
⋮	⋮
$x_{c}=x_{c,\max}\;\mathrm{Hz}$	$K\left[k\right]=\mathrm{DLQR}\;@x_{c,\max}\;\mathrm{Hz}$

**Table 2:** System constants.

Parameter	Value	Parameter	Value
*g*	9.80665 m/s^2^	*m*	0.515 kg
Q	$I_{10\times 10}q$	R	$I_{4\times 4}r$
$q={\left[130\;130\;130\;150\;150\;150\;2\;2\;2\;1\right]}^{T}$ $r={\left[180\;180\;180\;3\right]}^{T}$			
$K_{cp}=\left[1\;1\;1\;1\;1\;1\;1\;1\;1\;1\right]$ $\;K_{c}=1$			

**Table 3:** Evaluation metrics of different sampling and control strategies (the lower value denotes better performance).

Control Strategy	Physical				Computational	
	MSE	PSE	CE	W	CCT	SCT
Fixed-periodic	1.0000	1.0000	1.0000	1.0000	3.2609	2.7778
Event-triggered	2.1752	2.4783	1.0917	1.0426	1.0000	2.7778
Self-triggered	1.7726	2.3041	1.0778	1.0376	1.3478	1.1481
Co-regulation	1.0031	1.0270	1.0002	1.0001	1.1739	1.0000

**PSE**: physical state error; **MSE**: maximum state error; **CE**: control effort; **W**: energy cost; **SCT**: sampling computational time; **CCT**: control computational time

## Data Availability

Not applicable.
